# Study of the Thermodynamic Properties in Aqueous Solution of the Cyclocondensation Products of Pyrogallol and Propanaldehyde

**DOI:** 10.3390/molecules30193997

**Published:** 2025-10-06

**Authors:** Mauricio Maldonado, Diana Martínez, Almudena Crespo, Edilma Sanabria, Miguel A. Esteso

**Affiliations:** 1 Departamento de Química, Facultad de Ciencias, Universidad Nacional de Colombia, Sede Bogotá, Carrera 30 No. 45-03, Bogotá 111311, Colombia; mmaldonadov@unal.edu.co (M.M.); diamartinezra@unal.edu.co (D.M.); 2 Facultad de Ciencias y Artes, Universidad Católica de Ávila, Calle Los Canteros s/n, 05005 Ávila, Spain; almudena.crespo@ucavila.es; 3 Grupo GICRIM, Programa de Investigación Criminal, Universidad Manuela Beltrán, Avenida Circunvalar No. 60-00, Bogotá 111321, Colombia; edilma.sanabria@docentes.umb.edu.co; 4 Facultad de Ciencias de la Salud, Universidad Católica de Ávila, Calle Los Canteros s/n, 05005 Ávila, Spain; 5 Unidad Docente Química Física, Universidad de Alcalá, 28805 Alcalá de Henares, Spain

**Keywords:** pyrogallolarene, cyclocondensation, molecular association, *dimer capsules*

## Abstract

Pyrogallol[4]arenes are polyhydroxylated compounds obtained by condensation between pyrogallol and different aldehydes. Depending on both the type of aldehyde (aromatic or aliphatic) and the reaction time, these compounds can be obtained in different conformations, the most common being the *crown* and *chair* conformations. Using the conventional synthesis method, it is possible to obtain, in addition to the *chair* or *crown* conformers, other molecular associations, such as *dimer capsules*. The research in this study focuses on the synthesis products obtained from the condensation between pyrogallol and propanal. These products were characterized using spectroscopic methods, revealing that it is possible to obtain, in addition to the *crown* conformation, the *dimer capsule* of the macrocycle. Finally, the volumetric properties of these conformers were evaluated in dimethyl sulfoxide (DMSO) solution at several temperatures.

## 1. Introduction

Pyrogallol is a phenolic compound with three hydroxyl groups (-OH) located at the 1, 2, and 3 positions on a benzene ring, which gives it unique interaction properties with both metals and other molecules. In the early 20th century, phenolic compounds were well studied for their oxidation and reduction properties. The reaction between pyrogallol and aldehydes can produce macrocyclic systems known as calix[4]pyrogallolarenes. These polyhydroxylated platforms contributed significantly to the study of conformational flexibility in macrocyclic molecules. This was key to understanding how calixarenes, although initially considered rigid, can adapt to different shapes, giving them great versatility in their applications [1]. Calixarenes are polyhydroxylated compounds derived from the acid-catalyzed cyclocondensation reaction of phenolic compounds, such as pyrogallol and resorcinol, with aldehydes. This concept was introduced by C.D Gutsche in 1971, who investigated cyclic compounds with calix-like conformations. The discovery of these compounds was part of the development of organic chemistry of phenolic compounds and macrocyclic molecules which, throughout the 20th century, generated great interest due to their properties and their potential in applications of supramolecular chemistry [2,3]. The calix prefix was established for these molecules because the initially synthesized *t*-butyl-calixarenes presented a cup-like shape. However, it has recently been found that all condensation products between aldehydes and resorcinol or pyrogallol have this shape and therefore should be classified under this denomination. Therefore, it has been necessary to expand this classification, since a characteristic of *t*-butyl-calixarenes is their conformational flexibility, and many calixarene derivatives also exhibit diverse conformations [4].

Pyrogallol[4]arenes are synthesized by acid-catalyzed condensation between pyrogallol and an aldehyde. Currently, there are three methods for the synthesis of pyrogallol[4]arenes: one is based on transition metal-catalyzed condensation, the second method consists of a reflux method in a mineral acid, and the third by grinding the reactants [4]. The most common synthesis methods for the production of pyrogallolarenes use solvents such as ethanol, methanol, or solvent mixtures and involve long reaction times. In most cases, the reaction products are obtained by spontaneous precipitation in the reaction medium or it can be induced by increasing polarity [5].

The functionalization of pyrogallol[4]arenes consists of the structural modification of either the lower or the upper rim of the molecule in order to broaden the possible molecular interactions and selectivity with different analytes. This modification can be performed in two ways: first, during the synthesis of the macrocycle with a functionalized aldehyde that leads to the modification of the lower rim of the pyrogallol[4]arene; second, after the synthesis of the base pyrogallolarene, the functionalization is carried out on the hydroxyl groups (of the upper rim), enlarging the cavity of the pyrogallol[4]arene for further applications in host–guest complexations or assembly with other supramolecular systems [5]. Functionalization in the resorcinarenes and pyrogallolarenes at the upper rim occurs, for example, by an acylation reaction with acetic anhydride and pyridine [5]. In the case of functionalization at the lower rim, the synthesis allows changing the chain length linked to the methylene bridges by reaction with aliphatic aldehydes. It is also possible to introduce branched chains or chains containing a specific functional group [5,6]. Some research on the functionalization of pyrogallolarenes has mentioned the inclusion of both aliphatic and aromatics substituents [6], oxygenated groups, or groups that radically increase their polarity, making them soluble in water [7], and fluorophore groups that can generate changes in their spectroscopic properties [8].

Pyrogallol[4]arenes have a high electronic density due to their structure. This presents a cavity that allows interaction with different analytes; this characteristic offers the possibility of acting as a host system for the formation of host–guest complexes thanks to non-covalent molecular interactions such as cation-π, polar-π, and mainly CH-π interactions. Likewise, their structure allows interactions with the functional groups of the upper rim, which can influence the selectivity of the molecule [5]. These structural properties of the pyrogallolarenes have aroused great interest in recent years, and their versatility has been studied in a wide range of applications, such as their use in polymeric materials [9], heavy metal extraction agents [10], molecular filters [11], and others [12]. In their functionalized form, applications have been developed mainly as host–guest systems [1]. In this sense, the functionalization of pyrogallolarenes conformers has an important role, since the orientation of the functional groups present in the molecule varies the way they interact with other species and allows the formation of stable complexes with cations and anions [12,13,14]. For structural variant such as capsules, applications have been indicated as catalysts in biological processes [15], initiators for controlled polymerization [16], chemical receptors for ammonium ions in amino acids [17], ion transport through bilayer membranes [18], and binding of molecules to polyester surfaces [19]. The synthesis of these compounds also leads to the generation of mixtures of conformers or molecular aggregates, so the challenge of research is based on the search for the appropriate methodology to obtain the capsule conformation of an alkyl pyrogallolarene, ideally as the only conformer.

**Scheme 1:**
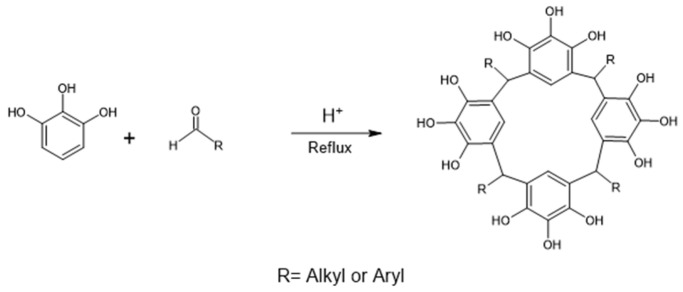
General reaction to obtain pyrogallol[4]arenes.

Continuing with the study of the reactivity of pyrogallol with aldehydes (Scheme 1) and the properties of pyrogallol[4]arenes [5,20,21], in the present article we show the reaction between pyrogallol and propanal to generate *C*-tetra(ethyl)pyrogallol[4]arene and the *dimer capsule* formed under the reaction conditions. The products thus obtained were characterized by using ESI-MS, FT-IR, and NMR spectroscopy. In addition, their volumetric properties were studied in dimethyl sulfoxide (DMSO) solution at several temperatures.

According to our review of the literature data about these products, this is the first time that a study of the thermodynamic properties of this type of derivative (the dimeric capsule) has been conducted. The results obtained in this work demonstrate the drastic change in its thermodynamic behavior.

## 2. Results and Discussion

### 2.1. Reaction of Propanaldehyde with Pyrogallol

**Scheme 2:**
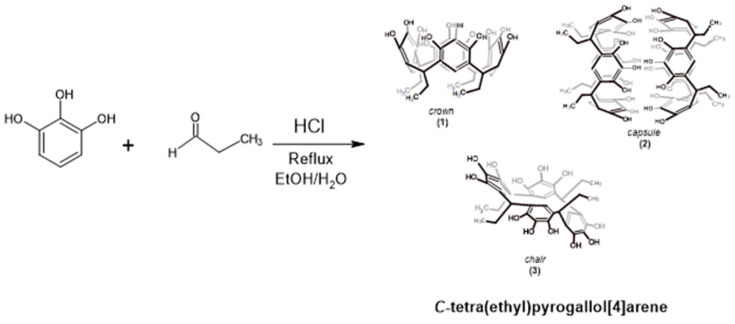
Cyclocondensation reaction of propanaldehyde with pyrogallol and its possible conformers.

The cyclocondensation reaction between propanaldehyde and pyrogallol was carried out according to the conventional method for obtaining these macrocycles [20], which is carried out in a mixture of ethanol and water (1:1) in an acidic medium under reflux conditions (Scheme 2). RP-HPLC analysis showed the formation of three products, after 4 h of reaction, which were isolated and purified by column chromatography as described in the experimental part. Table 1 shows the RP-HPLC characterization of the crude reaction.

Following this procedure, two products were isolated: the *crown* conformer of *C*-tetra(ethyl)pyrogallol[4]arene (product **1**) and the *dimer capsule* (product **2**). Product **3** could not be isolated due to its molecular dynamics.

**Figure 1:**
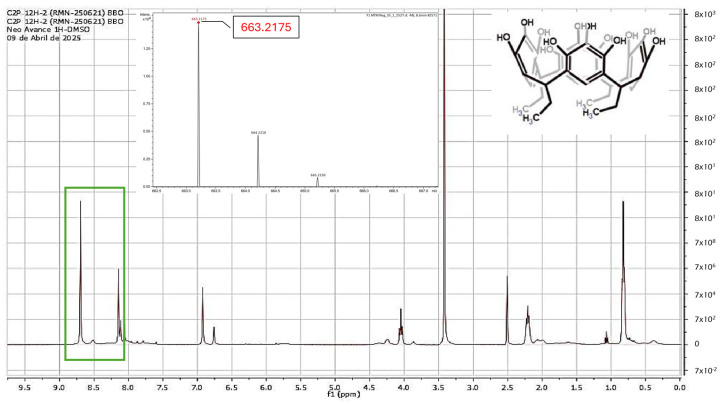
^1^H NMR and ESI-MS spectra of *C*-tetra(ethyl)pyrogallol[4]arene in *crown* conformation **(1)**.

The *crown* conformer (from now on, also called as the *monomer*) was characterized by different spectroscopic techniques. The FT-IR spectrum showed a band at 3272 cm^−1^ corresponding to the hydroxyl groups of the upper rim of the compound; three bands at (2955, 2980, and 2868) cm^−1^ characteristic of ethyl groups; and bands at (1621 and 1473) cm^−1^ indicating the presence of aromatic rings. The ^1^H NMR spectrum (Figure 1) showed a signal at 8.5 ppm and another at 8.7 ppm corresponding to the hydroxyl groups; the signal at 6.9 ppm is characteristic of the aromatic hydrogens; the triplet at 4.02 ppm corresponds to the methylene bridge between the aromatic rings; the signals at 2.20 ppm and at 0.81 ppm correspond to the protons of the ethyl chains. The ^13^C NMR spectrum in DMSO-d6 (Figure S5 in Supplementary Materials) showed four characteristic signals from the aromatic region (139.7; 132.9; 124.5; 113.6 ppm) and three signals from the ethyl chains which were observed at (36.2; 26.0; 12.9 ppm). Finally, the ESI-MS spectrum confirmed the formation of the *monomer* compund since the highest intensity peak was obtained at a ratio *m*/*z* = 663.2175 ([M − 1]), which matches the exact mass of the monomer (664.25 g/mol).

**Figure 2:**
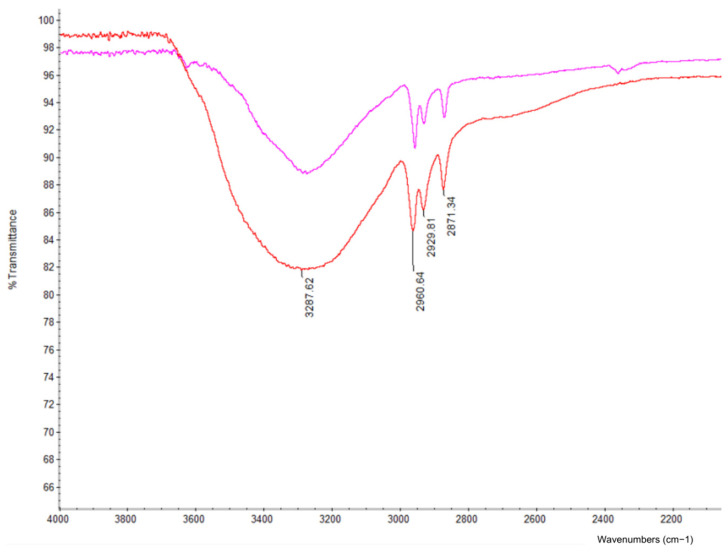
Comparison between the infrared spectra of the *crown* conformer **(1),** in purple color, and the *dimer capsule* **(2),** in red color.

The characterization of the second isolated product, the *dimer capsule* of *C*-tetra(ethyl)pyrogallol[4]arene **(2)** (from now on, also called as the *capsule*), in principle showed a great similarity with the results obtained for **(1)**. Regarding the FT-IR spectrum, the main difference was observed in the 3287 cm^−1^ band corresponding to the hydroxyls groups; as can be seen in Figure 2 this band is wider compared to that observed in the spectrum of **(1)**, possibly due to the interaction between the OH groups.

**Figure 3:**
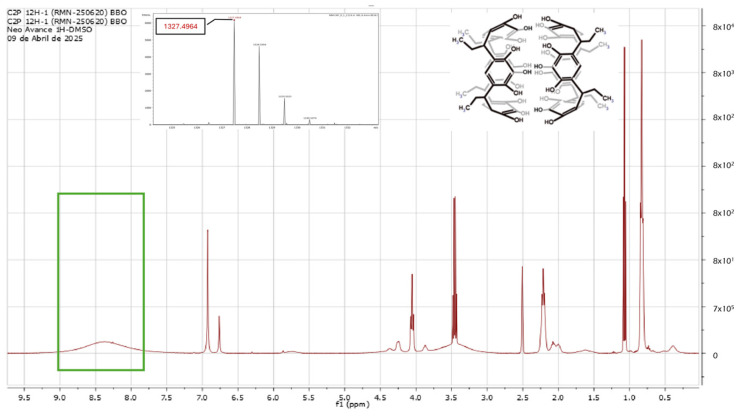
^1^H NMR and ESI-MS spectra of *dimer capsule* of *C*-tetra(ethyl)pyrogallol[4]arene.

On the other hand, the analysis performed on the ^1^H NMR spectrum (Figure 3) presented the same the signals in the high field, corresponding to the aliphatic region. According to the ^1^H NMR spectrum, two other signals also appear at 1.10 (t, J = 8 Hz) and 3.45 (q, J = 8 Hz) ppm, which were assigned to a methyl and methylene group, respectively, and confirm the presence of ethanol. In accordance with their integrals, it is evidenced that these are in 1:1 relation with the dimeric capsule **2**. This suggests that the ethanol molecule is encapsulated, due to the great tendency of these systems to occlude low molar mass molecules, as it has been shown in previous works on similar macrocycles [5,20]. The main difference to highlight is observed in the hydroxyl signals of these spectra, where that of the *capsule* **(2)** presents a broad singlet at 8.4 ppm which was integrated for 12 protons; the shape of this signal indicates that there is an intense interaction between the hydroxyl groups of the molecule. The signals observed in the ^13^C NMR spectrum in DMSO-d6 (see in Supplementary Materials) are very similar to those in the monomer spectrum **(1)**; in addition to these, signals were observed at 56.2 ppm and 18.7 ppm indicating the presence of ethanol, so the possibility that ethanol lodges in the cavity to form the dimer can be considered. To confirm this, ESI-MS characterization was performed, which showed the highest intensity peak at a ratio *m*/*z* = 1327.4964 ([M − 1]) a value consistent with the mass of the *capsule (m*/*z* = 1328.5); this corresponds to the expected signals for the *C*-tetra(ethyl)pyrogallol[4]arene and the expected structure for each pyrogallolarene according to similar studies [20,21,22].

### 2.2. Calculation of Apparent Molar Volume and Standard Molar Expansibility

From density measurements at different temperatures, apparent partial molal volume values were obtained using Equation (1) [23].

(1) $$V_{\phi }=\frac{M_{2}}{\rho }+\frac{1000\left(\rho_{0}-\rho \right)}{m\rho \rho_{0}}$$

where *M*_2_ is the molar mass of the solute, *ρ* and *ρ*_0_ correspond to the density of the solution and the solvent, respectively, and *m* is the molal concentration of solute.

The apparent partial molal volume values for dilute solutions of both pyrogallolarenes, *monomer* and *capsule*, in DMSO and at different temperatures are shown in Table 2 and Table 3, respectively. Uncertainties in apparent partial molal volume, due to uncertainty in density and molality, were estimated from the law of propagation of uncertainty [24]; these values were lower than ±0.80 cm^3^∙mol^−1^ for the solutions of the *monomer*, while for the *capsule* the uncertainties were smaller than ±1.27 cm^3^∙mol^−1^. The highest uncertainty values were found for the lowest concentrations.

**Figure 4:**
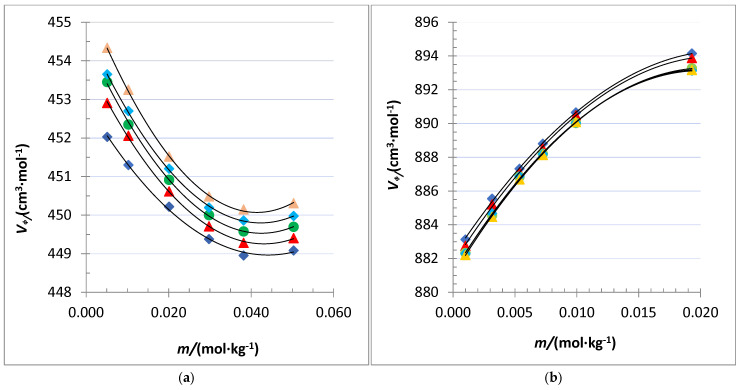
Dependence of the apparent partial molal volumes on the concentration at 293.15 K (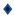), 298.15 K (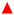), 303.15 K (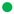), 308.15 K (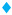), and 313.15 K (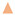) for: (**a**) *crown* pyrogallolarene, and (**b**) *dimer capsule* of pyrogallolarene.

The dependance of the apparent molal volumes with concentration, for the two conformers studied is shown in Figure 4a,b. This dependence was analyzed for the different temperatures studied by a least-squares fit to the quadratic empirical equation:

(2) $$V_{ɸ}=\;V_{2}^{o}+\;S_{v}m+\;B_{v}m^{2}$$

where the limiting value of the partial molal volume at infinitesimal concentration, $V_{ɸ,2}^{o}$ is equal to the standard molar volume value, $V_{2}^{o}$, $S_{v}$, and *B_v_* are experimental parameters reported in Table 4.

The variation of $V_{2}^{o}$ with *T* for both conformers was analyzed through the linear empirical relationship:

(3) $$V_{2}^{o}=a+\;bT$$

where *T* is the absolute temperature, and *a* and *b* are empirical parameters that were determined by fitting using the least squares method. Equation (3) was differentiated with respect to temperature to obtain the standard partial molar expansibility $E_{2}^{o}={\left(\;\partial V_{2}^{o}/\partial T\right)}_{p}=b$. The values of $V_{2}^{o}$, *S_v_*, *B_v_*, *a*, and *b* are shown in Table 4.

**Figure 5:**
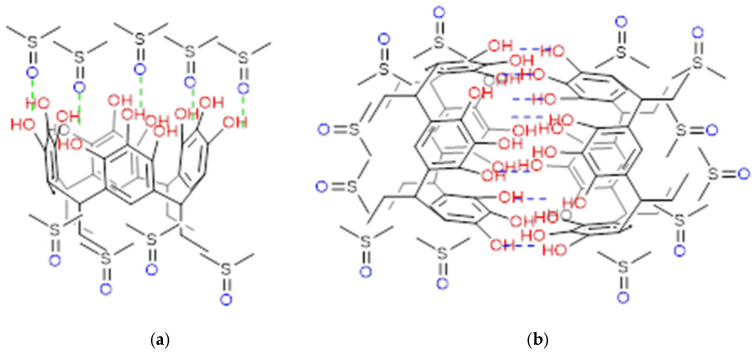
Representation of the interactions (hydrogen bonds and Van der Waals forces) between DMSO and: (**a**) *crown* conformer and (**b**) *dimer capsule* of *C*-tetra(ethyl)pyrogallol[4]arene.

As it can be observed, the *V_ϕ_* values obtained for the *monomer* in DMSO decrease with the molal concentration ($S_{V}$ < 0 at all temperatures), while for the *capsule*, under the same conditions, the *V_ϕ_* values increase with *m* ($S_{V}$ > 0 at all temperatures). In the first case, the results could indicate that as the concentration increases, the solute molecules are less solvated by the solvent, and that is why a smaller total volume is occupied by the solute and, therefore, a decrease in the apparent partial molal volume is observed. This may be due to the fact that the *monomer* is packaged in a more compact way as the concentration increases and, in this process, it expels the DMSO molecules that are on the sides and inside of the *monomer* conformer, which are not as strongly retained as those on the upper rim by interactions of the OH with the DMSO, which is an aprotic polar solvent. In the case of the *capsule*, an opposite behavior is observed, with an increase in the apparent partial molal volume as its concentration increases. This could be due to the fact that the capsule disrupts the structure of the DMSO, causing it to expand with the addition of more molecules. This could be because the DMSO can no longer interact with the OH groups on the upper rim of the pyrogallolarene, since they are no longer available when the capsule is formed (Figure 5a), thus breaking down the solvent. In both cases, as the concentration of the solute in the solution increases, solute–solute interaction also increases, and the apparent partial molal volume loses linearity with concentration.

On the other hand, because the apparent partial molal volume of the capsule is almost twice as large (i.e., 882.13 at 293.15 K) as that of the *monomer* (i.e., 452.92 at 293.15 K) at all temperatures studied, this behavior suggests that the conditions under which the synthesis reaction took place are suitable for the *monomer* conformer and the *capsule* of the macrocycle to be effectively separated.

Furthermore, the standard molar expansibility [25] is positive for the *monomer* ${(E}_{2}^{o}>0)$ which indicates that, with increasing temperature, either the solvation sphere increases or there is a structural relaxation of the solvent molecules around the solute molecules. Nevertheless, in the case of the *dimer capsule* pyrogallolarene, the standard molar expansibility is negative ${(E}_{2}^{o}<0)$, which implies that the molal volume decreases as the temperature increases (Table 3). This decrease could be due to two phenomena: a thermal contraction caused by a structural change in the solvent molecules around the solute molecules, which causes a rearrangement or compaction of the solvation sphere; or, more likely, that at higher temperatures some solvent molecules surrounding the capsule, which are not as strongly bound as in the case of the *monomer* alkylpyrogallolarene, leave the solvation sphere, causing the partial molal volume to decrease (Figure 5b).

## 3. Materials and Methods

IR spectra were recorded on a Nicolet iS10 FT–IR spectrometer (Thermo Fisher Scientific, Waltham, MA, USA) with a monolithic diamond ATR accessory, and peaks are reported in cm^−1^. ^1^H NMR spectra were recorded at 400 MHz, and ^13^C NMR spectra were recorded at 100 MHz on a Bruker Avance 400 instrument (Bruker Scientific Instruments, Billerica, MA, USA). Chemical shifts are reported in ppm, using the solvent residual signal. RP-HPLC analyses were performed over a Chomolith^®^ C18 column (Merck, Rahway, NJ, USA; 50 mm) using an Agilent 1200 liquid chromatograph (Agilent, Omaha, NE, USA), analysis was performed in gradient mode from 5 to 90% of solvent B (MeCN with 0.05% TFA, trifluoroacetic acid) in solvent A (water with 0.05% TFA, trifluoroacetic acid). The time gradient was 16 min. Detection was carried out at 210 nm and the flow rate was 2 mL/min. The sample concentration was 1.0 mg/mL, and the injection volume was 10 μL. The products were analyzed on a Bruker Impact II LC Q-TOF MS (Bruker Scientific Instruments, Billerica, MA, USA) equipped with electrospray ionization (ESI) in negative mode. The ESI source conditions were: End Plate Offset 500 V, Capillary 4500 V, Nebulizer 1.8 bar, Dry gas nitrogen 8.0 L/min, Dry Temp 220 °C. Scan mode AutoMS/MS with spectral range 20–1000 *m*/*z*, spectra rate 2 Hz, and collision energy of 5.0 eV.

### 3.1. Synthesis of C-tetra(ethyl)pyrogallol[4]arene

2.2 mmol of pyrogallol was dissolved in 20 mL of solvent mixture (EtOH/Water, 1:1) in a reaction balloon, then 1 mL of 37% hydrochloric acid was added dropwise, and this mixture was placed in an ice bath for 30 min. After this time, 11.9 mmol of propionaldehyde was added dropwise with stirring, and the mixture was taken to reflux at 80 °C with constant stirring. After the reaction time had elapsed, the solvent was evaporated in a rotary evaporator, and the products were subsequently purified by column chromatography, using silica gel as the stationary phase and variable mixtures of chloroform–methanol (90:10) as the mobile phase.

Tetra(ethyl)calix[4]pyrogallolarene (1): pink solid, mp > 250 °C; IR(ATR) υ/cm^−1^ 3272 (O-H, broad), 2955, 2928, 2868 (C-H, Ar, Aliph), 1621, and 1473 (-C=C, Ar); ^1^H NMR (400 MHz, DMSO-d6) δ 8.68 (s, 8H, OH); 8.13 (s, 4H, OH); 6.91 (s, 4H, Ar-H); 4.04 (t, J = 8 Hz, 4H, CH), 2.20 (q, J = 8 Hz, 8H, CH_2_); 0.81 (t, J = 8 Hz, 12H, CH_3_). ^13^C NMR (100 MHz, DMSO-d6) δ 139.7; 132.9; 124.5; 113.6; 36.2; 26.0; 12.9. ESI-MS: observed *m*/*z* = 663.2175 [M − 1]. Calcd. *m*/*z* = 663.22.

Dimeric Capsule of Tetra(ethyl)calix[4]pyrogallolarene (2): white solid, mp > 250 °C; IR(ATR) υ/cm^−1^ 3287 (O-H, broad), 2961, 2930, 2871 (C-H, Ar, Aliph), and 1614 (-C=C, Ar); ^1^H NMR (400 MHz, DMSO-d6) δ: 8.37 (br. s, 12H, OH); 6.92 (s, 4H, Ar-H); 4.05 (t, J = 8 Hz, 4H, CH), 2.21 (q, J = 8 Hz, 8H, CH_2_); 0.83 (t, J = 8 Hz, 12H, CH_3_). ^13^C NMR (100 MHz, DMSO-d6) δ 139.7; 133.0; 124.5; 113.6; 36.2; 26.0; 12.9. ESI-MS: observed *m*/*z* = 1327.4964 [M − 1]. Calcd. *m*/*z* = 1328.54.

### 3.2. Volumetric Properties of Monomer and Capsule Pyrogallolarene in DMSO Solution

The density of dilute DMSO solutions of *monomer* and *capsule* pyrogallolarene was measured between 293.15 K and 313.15 K, in five-degree intervals, using an Anton Paar, DSA 5000 M vibrating tube densimeter (Anton Paar Spain S.L.U., Madrid, Spain). The densimeter has a repeatability (s. d.) of ±1 × 10^−6^ g. cm^−3^. Its temperature repeatability (s.d.) is 0.001 degrees over both the temperature (0–100) °C and the pressure (0–116) psi ranges. The equipment was verified, as recommended by the supplier, with degassed Milli Q water (κ = 5.6 × 10^−8^ S cm^−1^) and air before each measurement series. From the density values, the apparent partial molal volumes, and by extrapolation of the limiting molar volumes, as well as their temperature dependences, were calculated. The values found for both conformers were compared and their behavior was analyzed.

The solvent used was dimethyl sulfoxide (DMSO) from Fluka (Honeywell, S.L., Madrid, Spain), with a mass fraction purity ≥ 0.99 (CAS number 67-68-5). The density values obtained for the different temperatures studied were consistent with those reported in the literature [26].

The purified solutes, both the *monomer* and the *capsule* pyrogallolarenes, were stored in a desiccator over silica gel until further use.

All solutions were prepared by weighing using a Sartorius analytical balance (Sartorius AG, Göttingen, Germany) whose uncertainty is 1 × 10^−5^ g in the range of interest. These solutions were degassed before use.

## 4. Conclusions

The *crown* tetra(ethyl)calix[4]pyrogallolarene and its *dimer capsule* were synthesized, isolated, purified, and characterized, obtaining acceptable yields after their separation by column chromatography. A detailed analysis of the ^1^H-NMR spectra of the two separated products confirmed that the signal pattern corresponds exclusively to the *crown*-type conformation for **1**. For the *dimer capsule* **2**, the spectroscopic signals showed that the two dimer units are also in *crown* conformation, and that they present broadening of the hydroxyl groups confirming the existence of a strong interaction at the upper rim of the *dimer capsule*. The formation of the *dimer capsule* was also confirmed by ESI-MS. Once the products were obtained, the density measurements performed on their DMSO solution showed a drastic change in the limiting partial molar volume $V_{2}^{o}$ for both products at the studied temperatures (between 293.15 K and 313.15 K).

## Figures and Tables

**Table 1:** HPLC analysis of the crude reaction of propanaldehyde with pyrogallol.

Conformer/Entry	Retention Time (min)	Yield (%) Solvent EtOH/H_2_O (1:1)
*Crown* **(1)**	10.3	(22%)
Dimer capsule **(2)**	10.7	(40%)
*Chair* **(3)**	9.9	(30%)

**Table 2:** Values of *ρ* and *V_ϕ_* for the *crown* conformer of *C*-tetra(ethyl)pyrogallol[4]arene (**1**) in DMSO in the temperature range from 293.15 to 313.15 K.

*T*/K	293.15 K		298.15 K		303.15 K		308.15 K		313.15 K	
*m*/ (mol∙kg^−1^)	*ρ*/ (g∙cm^−3^)	*V_ϕ_/* (cm^3^∙mol^−1^)	*ρ*/ (g∙cm^−3^)	*V_ϕ/_* (cm^3^∙mol^−1^)	*ρ*/ (g∙cm^−3^)	*V_ϕ/_* (cm^3^∙mol^−1^)	*ρ*/ (g∙cm^−3^)	*V_ϕ/_* (cm^3^∙mol^−1^)	*ρ*/ (g∙cm^−3^)	*V_ϕ/_* (cm^3^∙mol^−1^)
0.0050758	1.101341	452.03	1.096327	452.91	1.091316	453.45	1.086307	453.65	1.081298	454.34
0.010235	1.102292	451.34	1.097283	452.06	1.092280	452.35	1.087276	452.71	1.082273	453.23
0.020099	1.104116	450.22	1.099122	450.62	1.094131	450.92	1.089140	451.20	1.084151	451.52
0.029782	1.105906	449.38	1.100925	449.71	1.095946	450.00	1.090970	450.19	1.085993	450.49
0.038165	1.107444	448.95	1.102473	449.28	1.097504	449.58	1.092535	449.86	1.087568	450.15
0.050296	1.109618	449.08	1.104662	449.40	1.099708	449.70	1.094754	449.98	1.089799	450.31

Standard uncertainties are: *u*_r_(*m*) = 1.0·10^−3^ (max), *u*(*ρ*) = 0.150 kg·m^−3^, *u*(*V_ϕ_*) = 0.1 cm^3^·mol^−1^, and *u*(*T*) = 0.01 K.

**Table 3:** Values of *ρ* and *V_ϕ_* for the *dimer capsule* of *C-*tetra(ethyl)pyrogallol[4]arene (**2**) in DMSO in the temperature range from 293.15 to 313.15 K.

*T*/K	293.15 K		298.15 K		303.15 K		308.15 K		313.15 K	
*m*/ (mol∙kg^−1^)	*ρ*/ (g∙cm^−3^)	*V_ϕ_/* (cm^3^∙mol^−1^)	*ρ*/ (g∙cm^−3^)	*V_ϕ_/* (cm^3^∙mol^−1^)	*ρ*/ (g∙cm^−3^)	*V_ϕ_/* (cm^3^∙mol^−1^)	*ρ*/ (g∙cm^−3^)	*V_ϕ_/* (cm^3^∙mol^−1^)	*ρ*/ (g∙cm^−3^)	*V_ϕ_/* (cm^3^∙mol^−1^)
0.00099709	1.100766	883.13	1.095749	882.75	1.090732	882.33	1.085719	882.29	1.080707	882.21
0.0031561	1.101603	885.55	1.096593	885.21	1.091585	884.56	1.086578	884.68	1.081573	884.48
0.0053671	1.102448	887.32	1.097445	887.10	1.092443	886.85	1.087443	886.87	1.082445	886.69
0.0072539	1.103159	888.80	1.098163	888.53	1.093168	888.20	1.088174	888.19	1.083181	888.14
0.0099236	1.104152	890.65	1.099164	890.49	1.094180	890.03	1.089194	890.04	1.084208	890.10
0.019301	1.107575	894.15	1.102621	893.88	1.097675	893.26	1.092721	893.13	1.087763	893.18

Standard uncertainties are: *u*_r_(*m*) = 1.0·10^−3^ (max), *u*(*ρ*) = 0.150 kg·m^−3^, *u*(*V_ϕ_*) = 0.1 cm^3^·mol^−1^, and *u*(*T*) = 0.01 K.

**Table 4:** Values of $V_{2}^{o}$, *S_v_*, and *B_v_* for conformers *crown* and *dimer capsule* of pyrogallolarene in DMSO in the temperature range (293.15–313.15) K. The parameters of Equation (3), *a*, and $E_{2}^{o}(=b)$ are also shown.

Pyrogallolarene	*T*/K	$V_{2}^{o}$	$S_{v}$	*B_v_*
*Crown* (*monomer*)	293.15	452.92	−178.96	2027.7
	298.15	453.99	−219.79	2548.7
	303.15	454.53	−235.81	2778.8
	308.15	454.72	−233.36	2765.4
	313.15	455.60	−267.27	3227.8
	*a* = 424.02 (cm^3^∙mol^−1^)		${\;E}_{2}^{o}\left(=b\right)=\;$0.1004 (cm^3^∙mol^−1^∙K^−1^)	
*Dimer capsule*	293.15	882.13	1108.5	−25,197
	298.15	881.71	1147.8	−26,810
	303.15	881.19	1181.9	−28,845
	308.15	881.19	1191.1	−29,694
	313.15	881.01	1209.1	−29,971
	*a* = 898.24 (cm^3^∙mol^−1^)		${\;E}_{2}^{o}\left(=b\right)\;=$ −0.0552 (cm^3^∙mol^−1^∙K^−1^)	

$V_{2}^{o}$ in units of (cm^3^·mol^−1^), *S_v_* in units of (cm^3^·kg·mol^−2^), and *B_v_* in units of (cm^3^·kg^2^·mol^−3^).

## Data Availability

The data presented in this study are available on request from the corresponding author.

## Supplementary Materials

The following supporting information can be downloaded at: https://www.mdpi.com/article/10.3390/molecules30193997/s1, Figure S1. IR spectrum of C-tetra(ethyl)pyrogallol[4]arene (product **1**); Figure S2. IR spectrum of C-tetra(ethyl)pyrogallol[4]arene (product **2**); Figure S3. ^1^H NMR spectrum C-tetra(ethyl)pyrogallol[4]arene (product **1**); Figure S4. ^1^H NMR spectrum C-tetra(ethyl)pyrogallol[4]arene (product **2**); Figure S5. ^13^C NMR spectrum C-tetra(ethyl)pyrogallol[4]arene (product **1**); Figure S6. ^13^C NMR spectrum C-tetra(ethyl)pyrogallol[4]arene (product **2**); Figure S7. ESI-MS spectrum C-tetra(ethyl)pyrogallol[4]arene (product **1**); Figure S8. ESI-MS spectrum C-tetra(ethyl)pyrogallol[4]arene (product **2**).

## Abbreviations

FT-IR	Fourier Transform Infrared Spectroscopy
^1^H NMR	^1^H-Nuclear Magnetic Resonance
^13^C NMR	^13^C-Nuclear Magnetic Resonance
ESI-MS	Electrospray Ionization Mass Spectrometry
