# Supplementary material for: Study of the Thermodynamic Properties in Aqueous Solution of the Cyclocondensation Products of Pyrogallol and Propanaldehyde
Source: Molecules. 2025 Oct 6;30(19):3997. doi: 10.3390/molecules30193997 (PMC12526351; doi:10.3390/molecules30193997)

molecules

MDPI

Supplementary materials

Study of the Thermodynamic Properties in Aqueous Solution of the Cyclocondensation Products of Pyrogallol and Propanaldehyde

Mauricio Maldonado [1], Diana Martínez [1], Almudena Crespo [2], Edilma Sanabria [3] and Miguel A. Esteso [2,4]*

[1] Departamento de Química, Facultad de Ciencias, Universidad Nacional de Colombia, Sede Bogotá, Carrera 30 No. 45-03, Bogotá 111311, Colombia; mmaldonadov@unal.edu.co (M.M); diamartinezra@unal.edu.co (D.M)
[2] Universidad Católica de Ávila, Calle Los Canteros s/n, 05005 Ávila, Spain; almudena.crespo@ucavila.es (A.C.); mangel.esteso@ucavila.es (M.A.E.)
[3] Grupo GICRIM, Programa de Investigación Criminal, Universidad Manuela Beltrán, Avenida Circunvalar No. 60-00, Bogotá 111321, Colombia; edilma.sanabria@docentes.umb.edu.co
[4] U.D. Química Física, Universidad de Alcalá, 28805 Alcalá de Henares, Spain
* Correspondence: mangel.esteso@ucavila.es

Table of Contents:

Figure S1. IR spectrum of C-tetra(ethyl)pyrogallol[4]arene (product **1**) 2
Figure S2. IR spectrum of C-tetra(ethyl)pyrogallol[4]arene (product **2**) 3
Figure S3. ^{1}H NMR spectrum C-tetra(ethyl)pyrogallol[4]arene (product **1**) 4
Figure S4. ^{1}H NMR spectrum C-tetra(ethyl)pyrogallol[4]arene (product **2**) 5
Figure S5. ^{13}C NMR spectrum *C*-tetra(ethyl)pyrogallol[4]arene (product **1**)................................6
Figure S6. ^{13}C NMR spectrum *C*-tetra(ethyl)pyrogallol[4]arene (product **2**)................................7
Figure S7. ESI-MS spectrum *C*-tetra(ethyl)pyrogallol[4]arene (product **1**) 8
Figure S8. ESI-MS spectrum *C*-tetra(ethyl)pyrogallol[4]arene (product **2**) 9

Figure S1. IR spectrum of *C*-tetra(ethyl)pyrogallol[4]arene (product **1**)

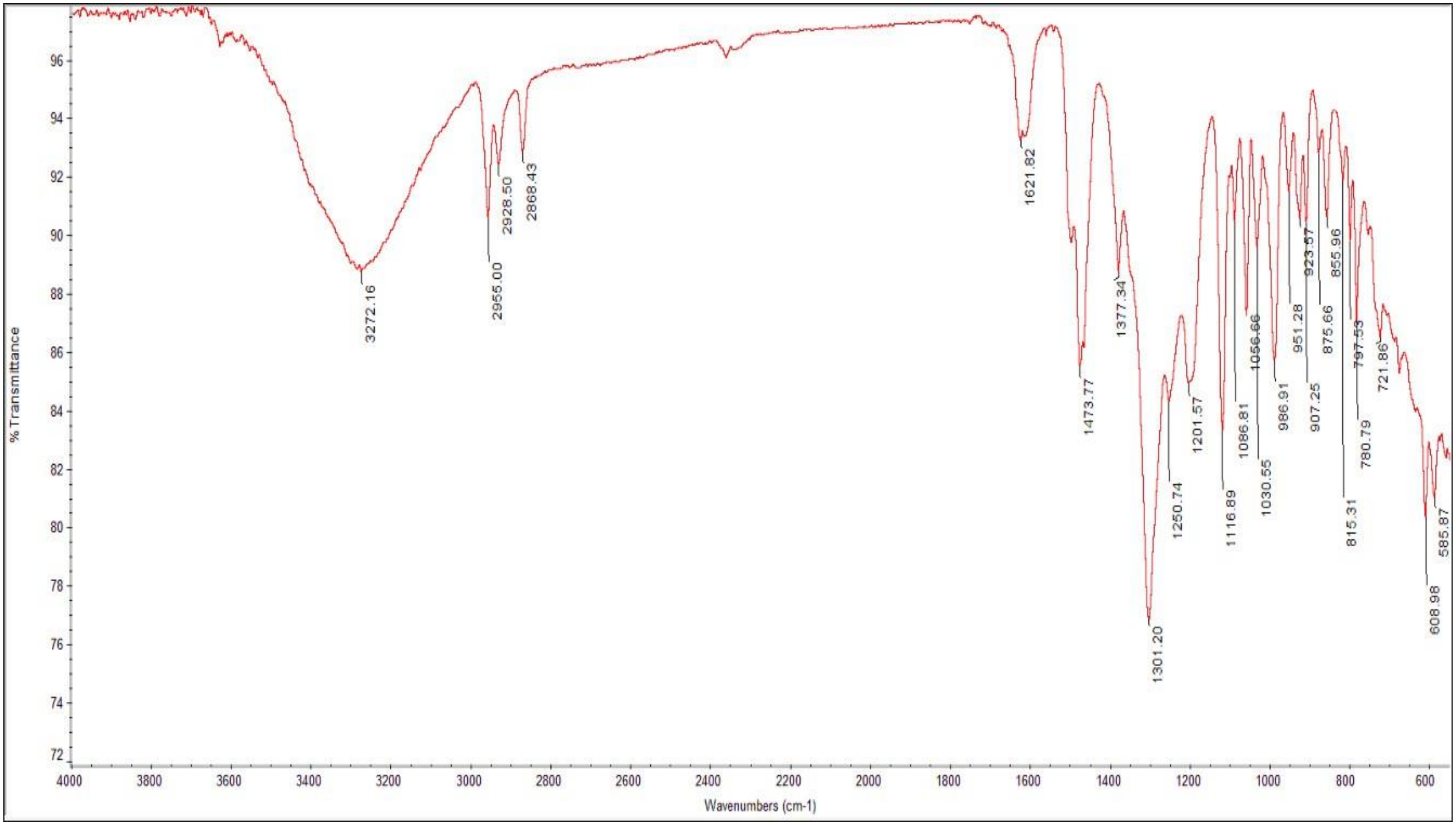

Figure S2. IR spectrum of *C*-tetra(ethyl)pyrogallol[4]arene (product 2)

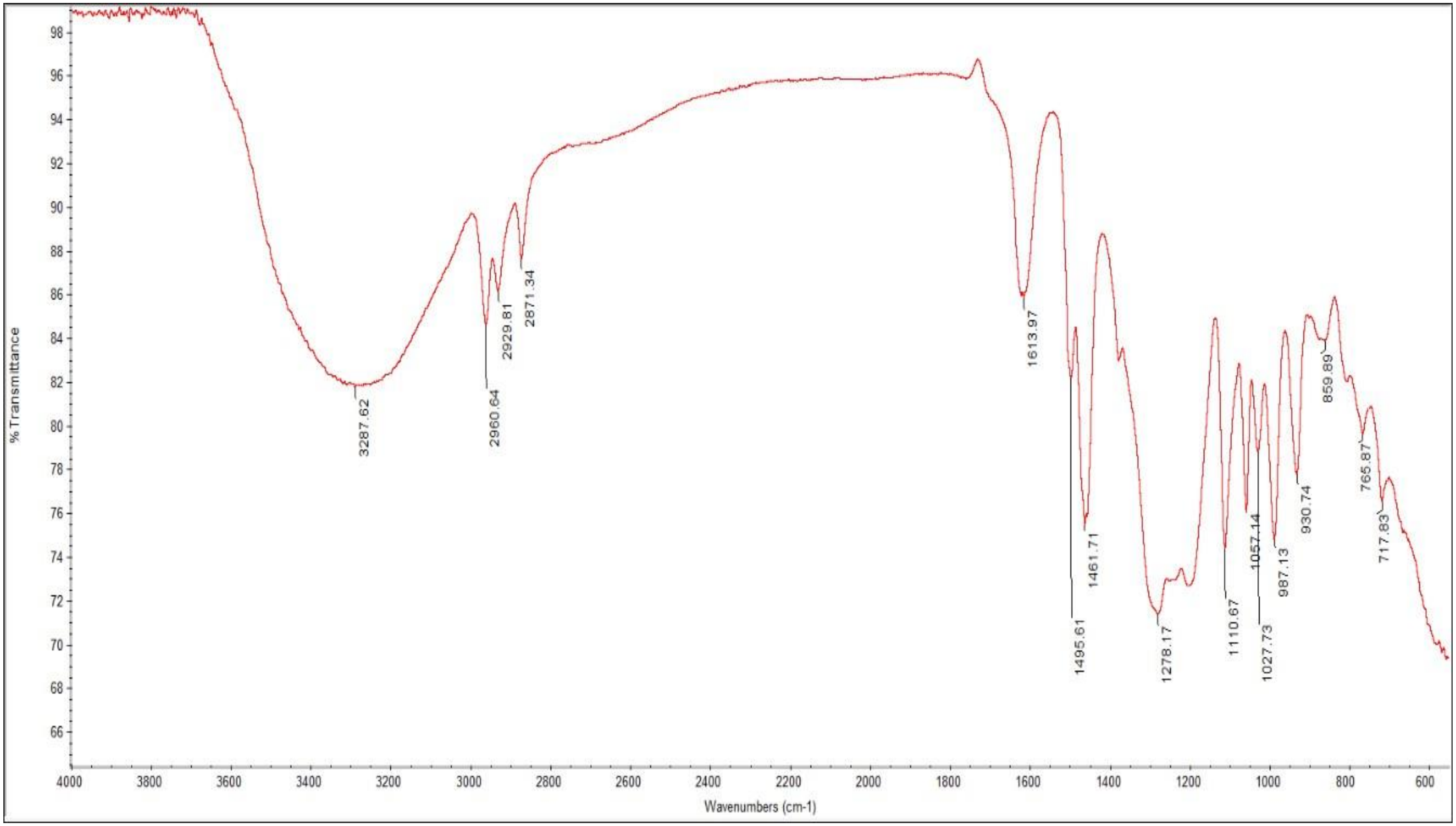

Figure S3. ¹H NMR spectrum of *C*-tetra(ethyl)pyrogallol[4]arene (product 1)

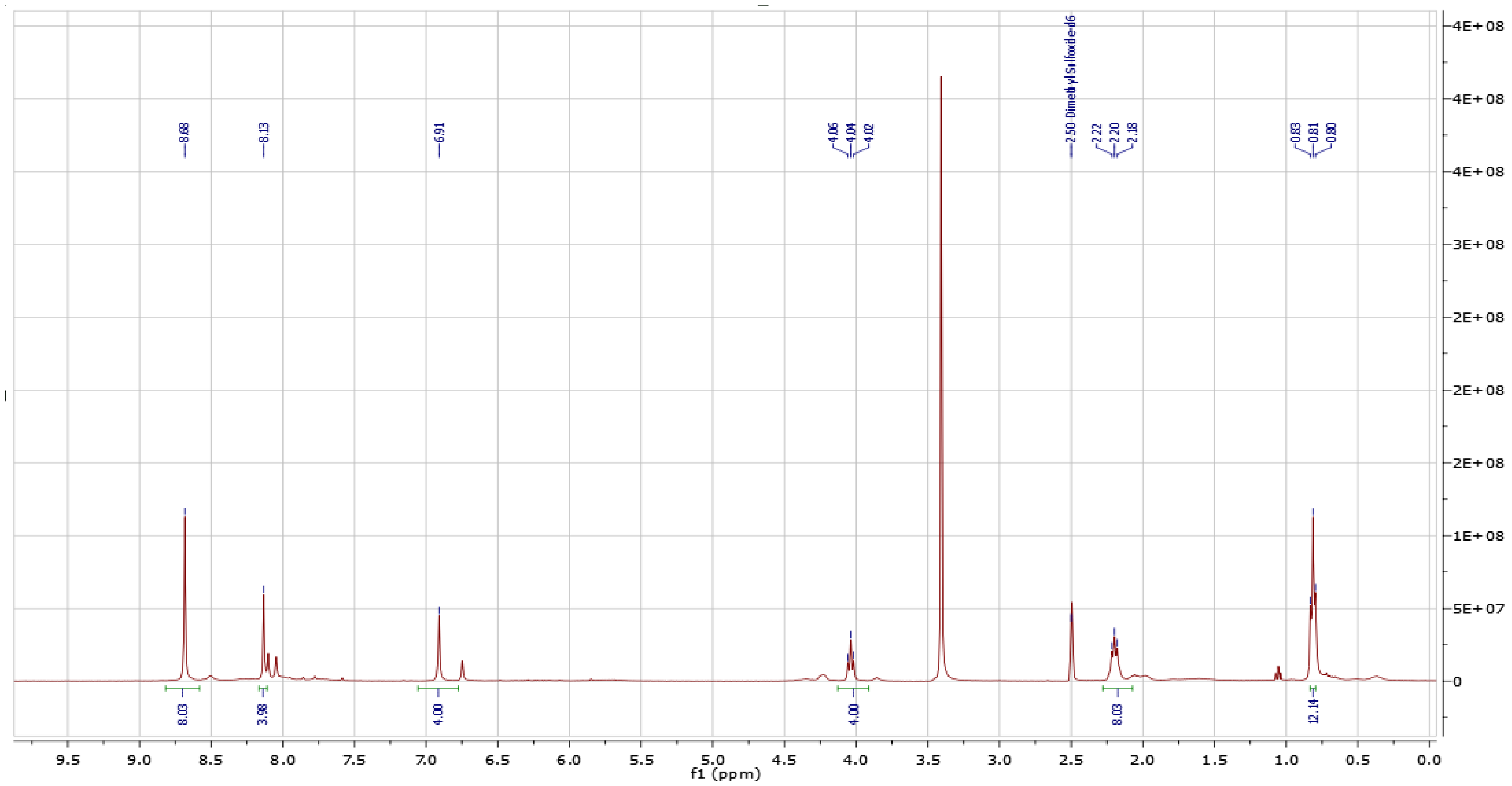

Figure S4. ^{1}**H NMR spectrum of** ***C*****-tetra(ethyl)pyrogallol[4]arene (product 2)**

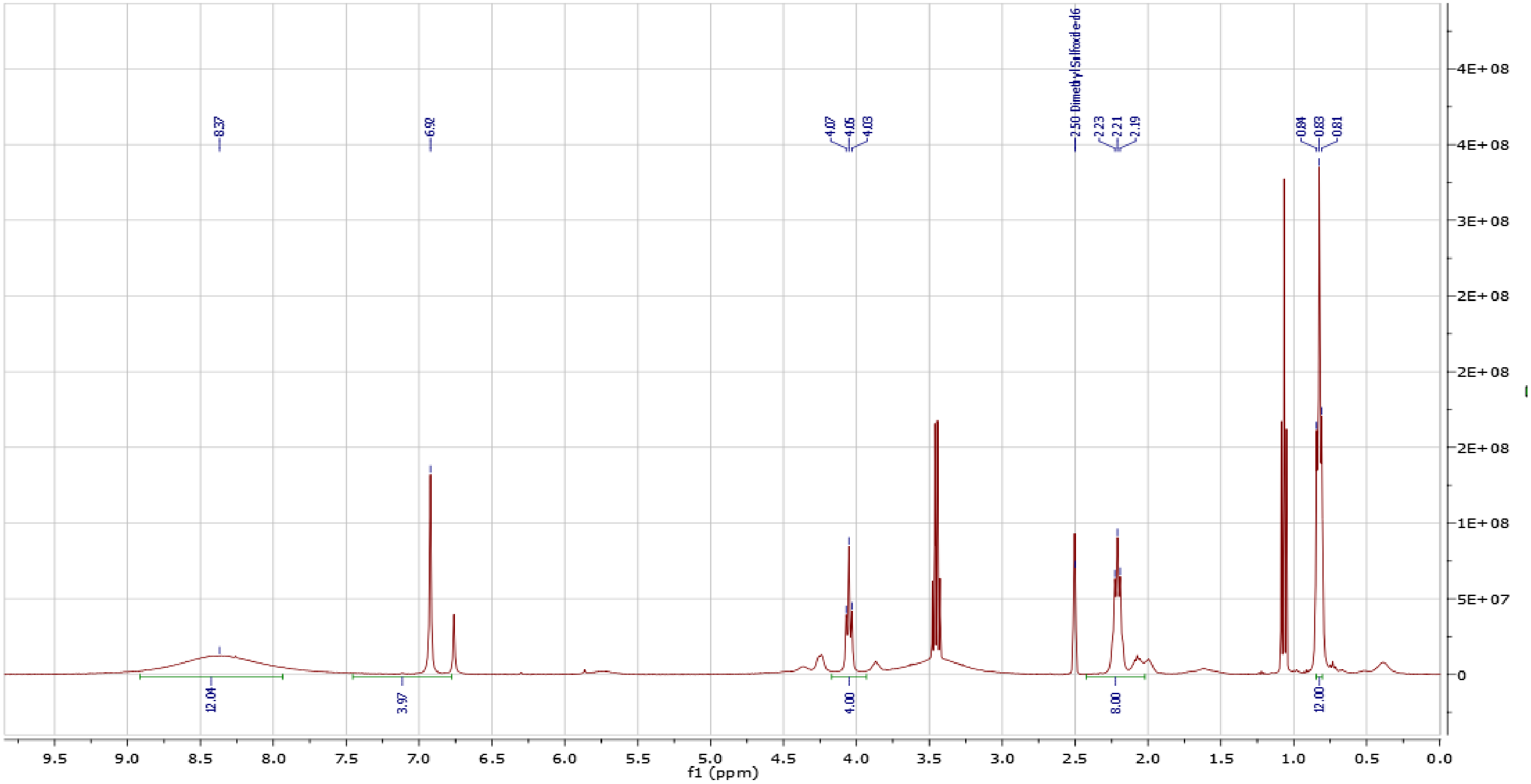

Figure S5. ^{13}C **NMR spectrum of *C*-tetra(ethyl)pyrogallol[4]arene (product 1)**

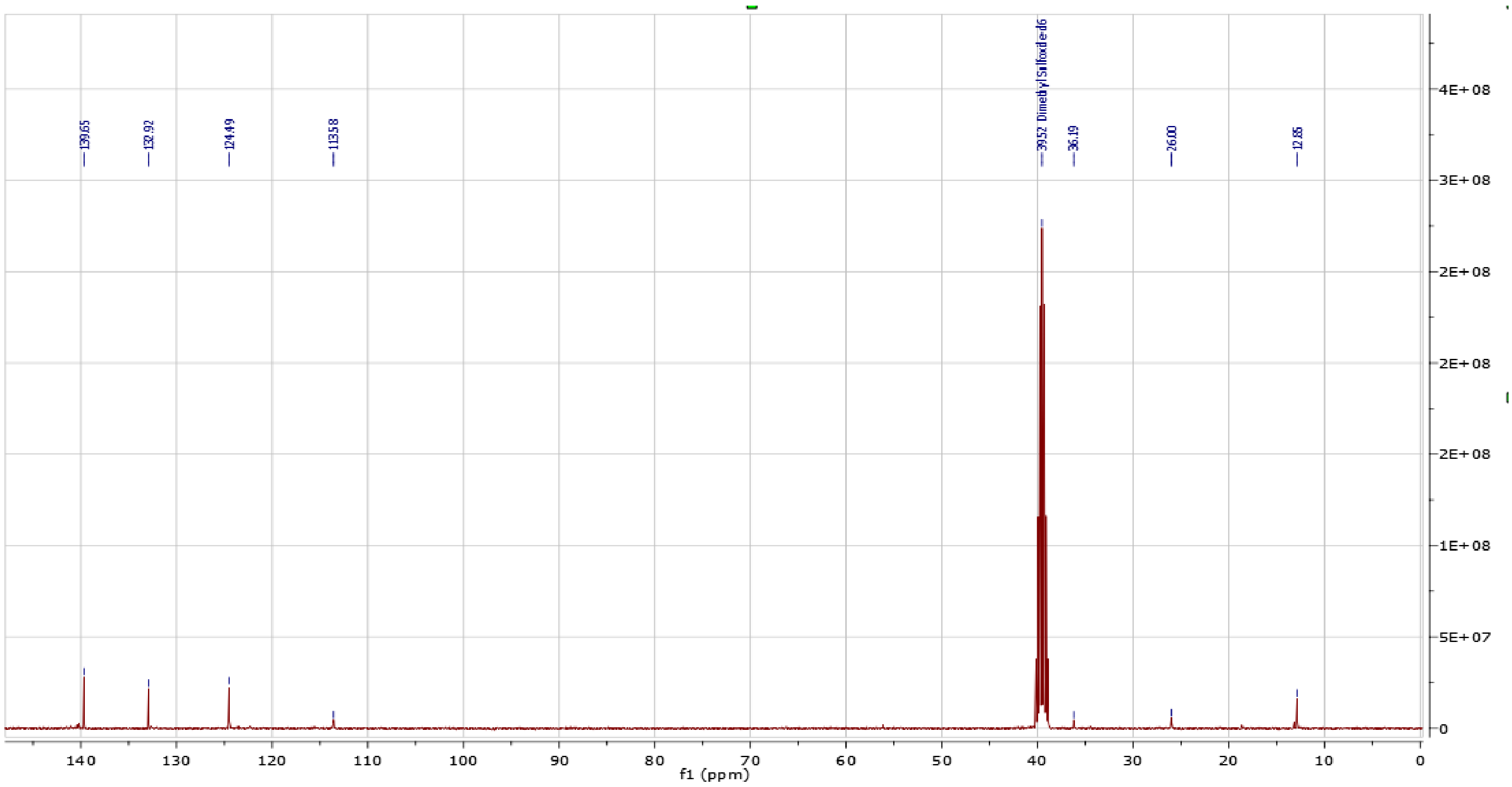

Figure S6. ^{13}C NMR spectrum of *C*-tetra(ethyl)pyrogallol[4]arene (product 2)

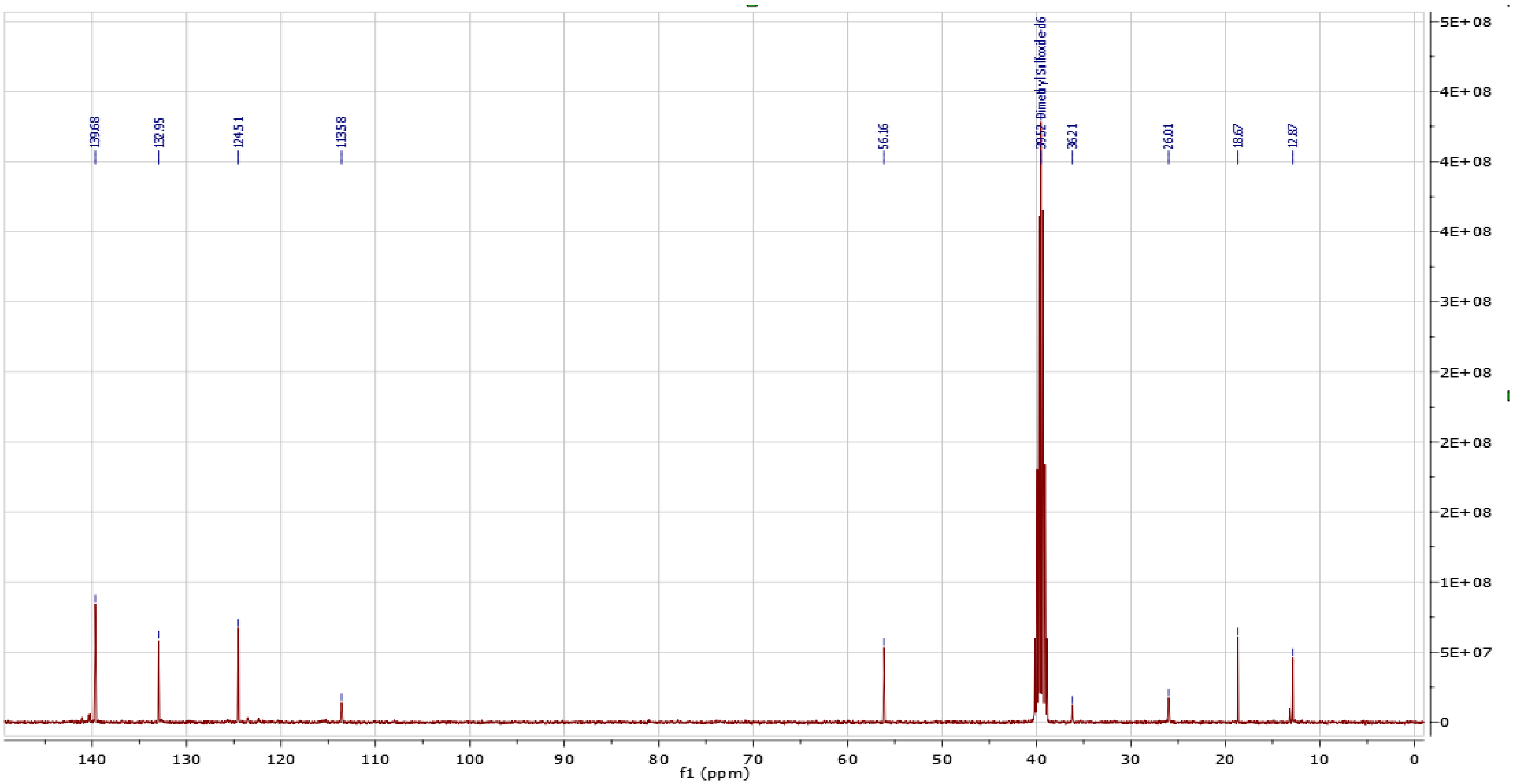

Figure S7. ESI-MS spectrum of *C*-tetra(ethyl)pyrogallol[4]arene (product 1)

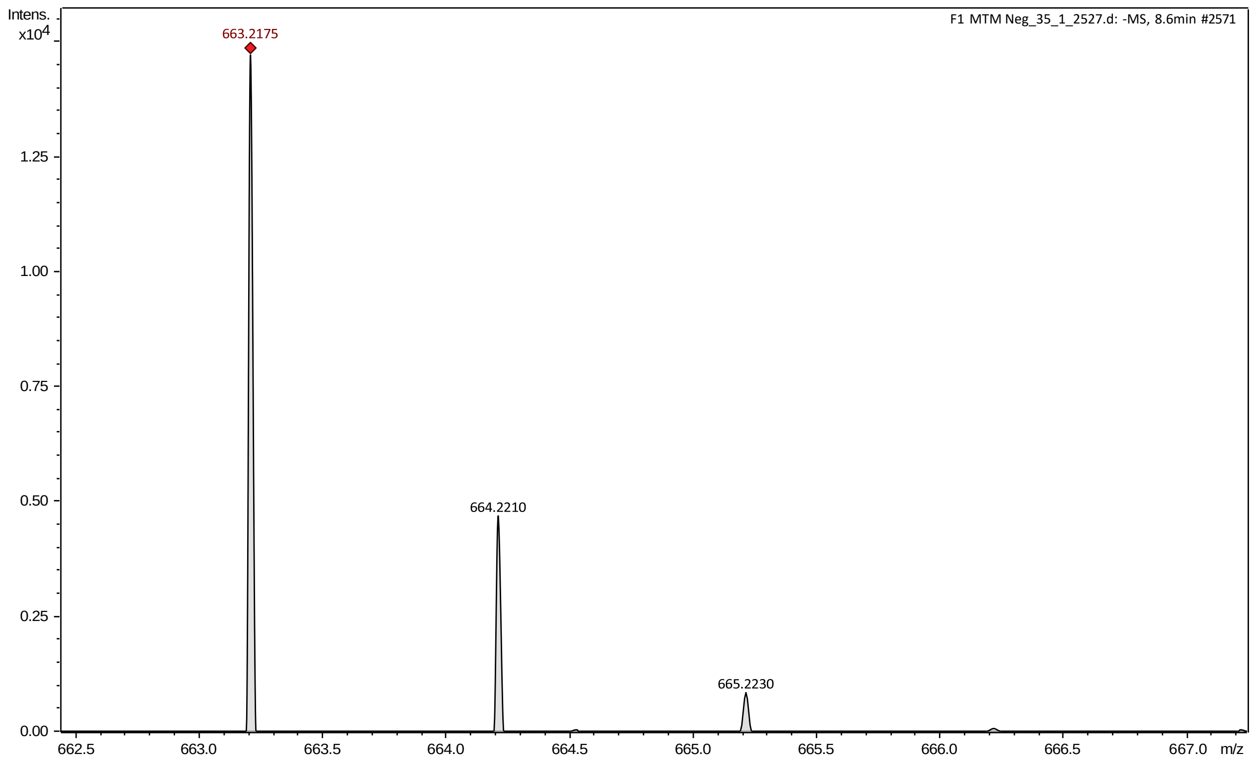

Figure S8. ESI-MS spectrum of *C*-tetra(ethyl)pyrogallol[4]arene (product 2)

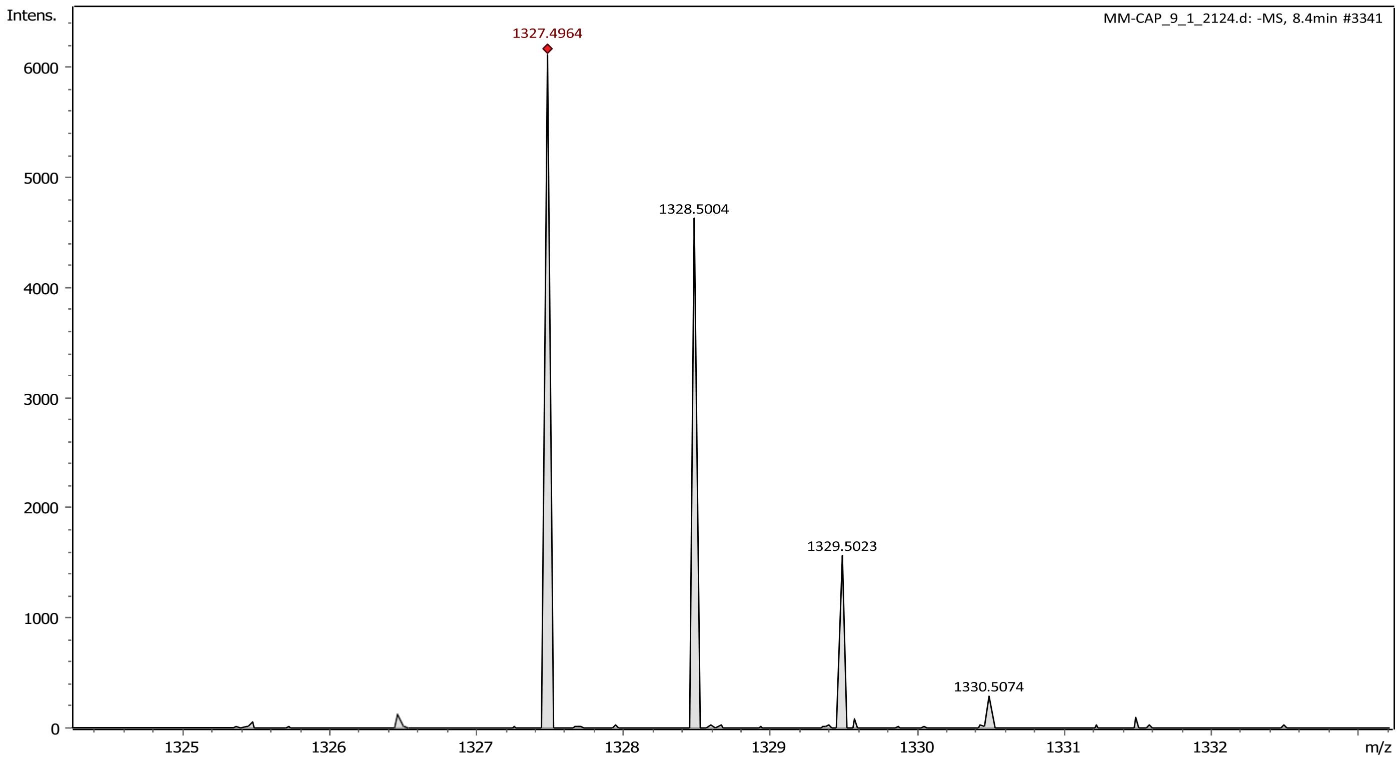

Supplement: Supplementary file 1 [file molecules-30-03997-s001.zip › molecules-3849962-supplementary.pdf]
